# Direct Laser Interference Patterning for Wettability Modification and Bubble Nucleation on Conventional and Additively Manufactured Metals

**DOI:** 10.1021/acs.langmuir.6c00056

**Published:** 2026-03-27

**Authors:** Julian Heinrich, Fabian Ränke, Karin Schwarzenberger, Tine Marquardt, Xuegeng Yang, Mateusz Marek Marzec, Krystian Sokołowski, Andrés Fabián Lasagni, Kerstin Eckert

**Affiliations:** † Institute of Fluid Dynamics, 28414 Helmholtz-Zentrum Dresden-Rossendorf, Bautzner Landstr. 400, 01328 Dresden, Germany; ‡ Institute of Process Engineering and Environmental Technology, 9169 Technische Universität Dresden, Helmholtzstr. 10, 01069 Dresden, Germany; § Institute of Manufacturing, Technische Universität Dresden, George-Baehr-Str. 3c, 01069 Dresden, Germany; ∥ Academic Centre for Materials and Nanotechnology, AGH University of Krakow, Av. Mickiewicza 30, 30-059 Krakow, Poland; ⊥ Fraunhofer Institute for Material and Beam Technology IWS, Winterbergstraße 28, 01277 Dresden, Germany

## Abstract

In addition to surface chemistry, surface roughness plays a critical role regarding wettability and solid–liquid as well as solid–gas interactions. Additive manufacturing produces substrates with unique characteristics (such as inherent roughness and porosity) that differ significantly from those of conventionally fabricated materials. In this study, conventionally manufactured Ti64 and stainless steel 316L substrates are compared with additively manufactured stainless steel 316L in their as-fabricated state, as well as after the application of direct laser interference patterning to introduce additional micro- and nanostructures. Surface morphology and topography are characterized using confocal microscopy and scanning electron microscopy. Wettability development is evaluated after storage in ambient air and aqueous environments, and the observed behaviors are correlated with different wetting states. Furthermore, the influence of these wetting states on bubble dynamics in O_2_-oversaturated aqueous solutions is investigated. The results indicate that the intrinsic roughness of AM substrates significantly enhances gas nucleation, primarily due to increased surface area and the presence of Harvey nuclei. Additional laser structuring by direct laser interference patterning not only increases the surface area but also oxidizes the surface and can induce rapid changes in surface chemistry, thereby affecting solid–gas interactions. Notably, the laser treatment of Ti64 substrates led to the formation of surfaces with very high water contact angles, characterized by the rose petal wetting regime. Despite the apparent superhydrophobic character, these surfaces did not promote solid–gas interactions. Other obtained wetting states turned out to be more beneficial for enhancing bubble nucleation. This work underscores the complex interplay between surface topography and chemical modification in achieving specific wetting states and highlights their collective impact on solid–gas interfacial phenomena.

## Graphical abstract

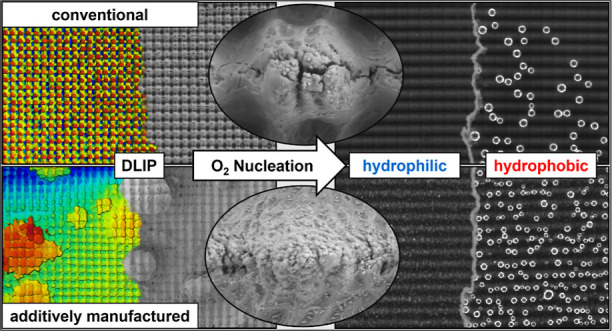

## Introduction

Bubble nucleation and growth within oversaturated solutions are common phenomena in a wide range of natural and industrial processes, e.g., boiling of superheated liquids, carbonated beverages or electrolysis.^1–3^ For nucleation to take place, i.e., the formation of a gas bubble inside a liquid, an energy barrier must be overcome in the form of the surface energy required to create the new gas–liquid interface.^1^ According to Jones et al., bubble nucleation can be categorized into four distinct types:^2^ Type I refers to classical homogeneous nucleation, which occurs within the bulk liquid in the absence of any solid surfaces. Type II corresponds to classical heterogeneous nucleation events, occurring on a solid–liquid interface, present by impurities or other solid surfaces inside the liquid, but without pre-existing gas cavities on the solid surface. Type III describes pseudoclassical nucleation, in which bubbles form at pre-existing gas cavities known as Harvey nuclei. Those cavities are smaller than the critical radius *r*_crit_ and are activated by local fluctuations in oversaturation. Lastly, Type IV represents non-classical nucleation involving pre-existing gas cavities already greater than *r*_crit_. Especially Types II to IV play major roles in industrial processes which include transition of dissolved components into their own gaseous phase. In these cases, the presence of a solid surface reduces the nucleation energy barrier by decreasing the critical nucleation radius, owing to a reduced liquid–gas interfacial area. Furthermore, increased surface roughness promotes nucleation by providing a greater number of Harvey nuclei.^2,3^

The energy barrier is strongly influenced by both surface roughness and chemistry. These factors also govern—and are effectively characterized by—surface wettability, which describes the spreading of a liquid on a solid based on intermolecular interactions.^4,5^ At equilibrium conditions a water droplet stabilizes at a characteristic static water contact angle (WCA), also known as the Young contact angle θ_*y*_ (only applies for ideal surfaces, i.e., smooth, chemically homogeneous, rigid, insoluble, non-reactive, flat).^6,7^ It is visualized in Figure 1a, including the interfacial energy γ at the different interfaces (solid–gas (SG), gas–liquid (GL) and solid–liquid (SL). The term γ_SG_ also represents the total surface free energy (SFE) relating to homogeneous and smooth surface conditions.^8,9^ In this context, chemical bonds at the surface influence interfacial solid–liquid interactions: in case of dominating adhesive forces at the solid–liquid interface, the liquid spreads easily. Conversely, if the cohesive forces within the liquid exceed the adhesive forces at the interface, the liquid tends to minimize its contact area with the solid, leading to partial or non-wetting.^5,7^ Typically, the maximum WCA of a flat and smooth surface is in the order of 120°.^10^ Non-ideal surfaces can reach higher contact angles due to surface roughness, represented by two classical models: Wenzel, describing a completely wetted two-phase solid–liquid interface over the roughness features,^11^ and Cassie–Baxter, describing a non-homogeneous regime with a three-phase solid–liquid–gas interface with trapped air pockets,^12^ see Figure 1c,d. Each model incorporates surface roughness in a distinct way. Wenzel relates the measured apparent WCA θ* to the Young WCA θ_*y*_ using the solid roughness factor r.^7,11^ In the Cassie–Baxter state, an additional parameter *f*, representing the fractional projected solid–liquid contact area beneath the droplet, is required to describe the system.^7,12^

**Figure 1:**
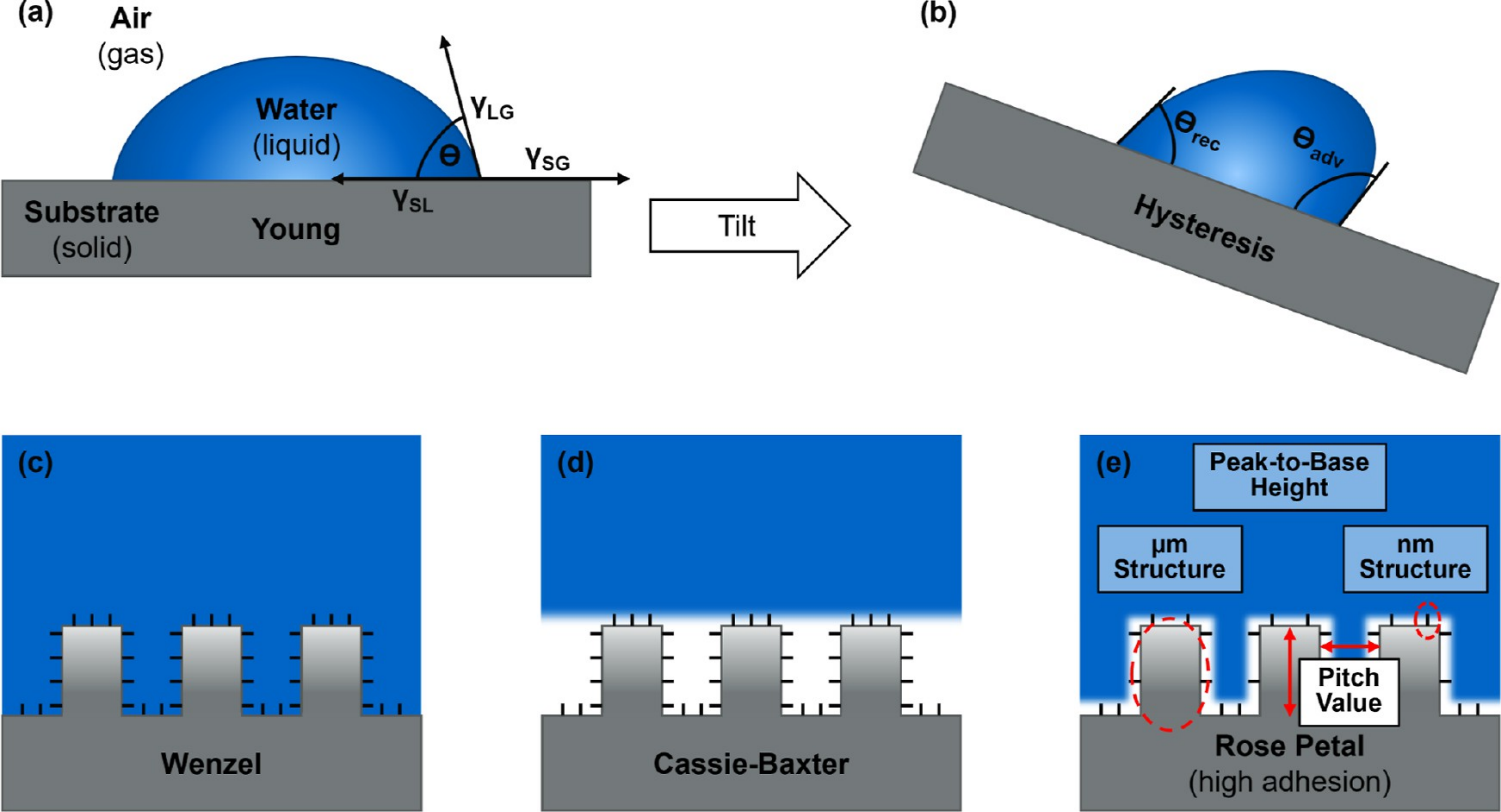
Surface wetting according to (a) Young’s model on a smooth and ideal surface, (b) contact angle hysteresis (CAH), (c) Wenzel state on a hierarchical surface structure, (d) Cassie–Baxter state with entrapped air pockets between microstructures and (e) rose petal effect with impregnated microstructures but only partially wetted nanostructures.

However, both models have inherent limitations and do not account for dynamic wetting phenomena like contact angle hysteresis (CAH), arising from energy dissipation, i.e., the difference between the energy gained when the liquid comes into contact with the solid surface and the energy recovered upon separation (typically being smaller). The CAH is described by the difference between the advancing angle θ_adv_ and the receding angle θ_rec_ (see Figure 1b). Factors that affect CAH include adhesion, surface roughness and inhomogeneity.^13,14^ To account for hysteresis effects, additional models have been proposed. Some focus especially on superhydrophobic surfaces (while a debatable term, generally referring to WCA > 150° ^15–17^ ): namely the well-known lotus effect^18^ and the more recently discovered rose petal effect.^19^ The lotus effect is based on a hierarchical surface texture promoting a superhydrophobic behavior combined with low adhesion and small CAH. On the contrary, the rose petal effect (displayed in Figure 1e), despite the overall high WCA, exhibits strong adhesion of water leading to large CAH which can usually be attributed to a larger solid–liquid contact area and contact line pinning effects.^13,14,19,20^ Those pinning effects on structurally highly heterogeneous surfaces arise from local minima in the Gibbs energy curve along the apparent WCA of the droplet. Therefore, an energy barrier must be overcome to transition from one minimum to another.^17,21^ Regarding the different CAH behaviors of lotus and rose petal effect, Bhushan et al.^14^ proposed these variations are influenced by the characteristics of the hierarchical structures. These characteristics refer to the ratio between the pitch value (i.e., distance between microstructures), their peak-to-base height and the nanostructure density. Surfaces characterized by a small pitch value, a high peak-to-base height, and a high density of nanostructures promote the formation of air pockets (i.e., Cassie–Baxter wetting regime). The water droplet rests on the top of the surface asperities while air remains trapped within the underlying surface structure. This reduces the effective solid–liquid contact area, resulting in high static WCAs, low CAH, and low adhesion. Conversely, a large pitch value in combination with a small peak-to-base height and less nanostructures would allow the water to impregnate between the microstructures (i.e., Cassie impregnating wetting regime). However, the nanostructures remain incompletely wetted, causing high CAH and high adhesion (see Figure 1e). For non-ideal surfaces several approaches like the normalized surface free energy (NSFE)^22^ or corrections via the Wenzel equation^9^ are suggested to relate the measured WCA of different test liquids to the SFE. However, these approaches typically neglect that several metastable states in the measured apparent contact angle of rough surfaces exist which lie between the advancing and receding contact angle, and thus no clear relation between the measured contact angles of the test liquids and any kind of surface free energy value can be derived.

Since both surface chemistry and surface roughness significantly influence wettability, they are also closely related to the modulation of solid–gas interactions, particularly in the context of heterogeneous nucleation (Type II to IV). Increased surface roughness facilitates the presence of Harvey nuclei and therefore enhanced nucleation of Type III and Type IV. For example, it has been observed that rose petal structures not only pin droplets on their surface but also bubbles when immersed in water. Air pockets retained within the nanoscale features of the surface can coalesce with adjacent bubbles, anchoring them to the substrate through contact line pinning.^23^ Therefore, highly rough surfaces may offer untapped potential for improving solid–gas interactions and increasing nucleation site density.

Additively manufactured (AM) metal substrates inherently possess a high degree of surface roughness,^24^ offering distinct characteristics compared to conventionally manufactured materials. While post-processing steps such as laser polishing are commonly used to reduce roughness and homogenize the surface topography by remelting the outer layer,^25,26^ only a limited selection of processes can be used to apply additional structures to the substrate. Direct laser interference patterning (DLIP) enables such implementations and the tailoring of surface functionalities. DLIP is based on the interference of multiple coherent laser beams in the focal plane, resulting in an interference pattern characterized by a periodic spatial distribution of laser intensity. The geometry of this interference pattern is determined by several parameters^27^ and allows to fabricate a wide variety of surface structures.^28^ By employing ultrashort laser pulses, energy deposition can be confined to the interference maxima for localized heating of the material. This minimizes the formation of undesired molten zones and facilitates the generation of highly uniform surface patterns, even at sub-micrometer scales.^29^ A key advantage of DLIP lies in its ability to simultaneously produce a large number of periodic features within a single laser pulse. Typically, the resulting structures exhibit lateral dimensions ranging from approximately 0.5–30.0 μm. Due to the utilization of ultra short laser pulses in the ps or fs regime, self-organizing features called laser-induced periodic surface structures (LIPSS) are formed simultaneously, enabling the fabrication of hierarchical surface topographies which further increase the available surface area.

In recent years, DLIP has been successfully employed for the functionalization of AM metal components,^30^ owing to its capability for precise surface structuring even on substrates exhibiting high roughness and significant surface irregularities. Furthermore, it modifies surface wettability by inducing changes in both surface topography and chemical composition.^31,32^ In principle, the laser treated metallic surfaces initially demonstrate a hydrophilic wetting behavior coupled with a high SFE, attributed to the formation of a superficial metal oxide layer with highly energetic bonds during laser processing under ambient conditions.^7,27–29^ The generated metal oxides display a high degree of polarity due to the large difference in electronegativity between the atoms and are usually of polar or ionic nature.^33^ Current theories suggest that the laser process leads to a temporary deficit of surface electrons, facilitating hydrogen bonding with interfacial water molecules.^7^ Depending on the post-treatment storage conditions, this hydrophilicity can partially be preserved.^7,27^ However, upon prolonged exposure to ambient air, adsorption processes of organic molecules take place, causing a hydrophobization of the surface layer.^7^

By changing the process parameters, different surface geometries can be achieved and the degree of micro- and nanostructures can be varied. Previous studies have demonstrated that aspect ratios of approximately 1 have a stronger influence on the surface character compared to a shallower topography.^30,34,35^ To extend these findings, this study aims to generate even deeper structures with an aspect ratio >1, further applying them on AM materials to combine the structuring process with the intrinsic roughness. The DLIP applicability on AM materials will be analyzed, as well as its effectiveness to additionally enhance solid–gas interactions, i.e., nucleation site density and bubble growth. Furthermore, the wetting properties will be characterized for two storage environments—ambient air and deionized (DI) water—because post-treatment changes in surface chemistry are expected to differ depending on the surrounding medium.

## Materials and Methods

### Materials

Metal sheets with a size of 10.0 × 10.0 × 1.0 mm were used as substrate materials. Two types of conventional materials were employed: Ti64 (90 wt % titanium, 6 wt % aluminum, 4 wt % vanadium, Goodfellow) and SS 316L (Versand Metall). To ensure uniform roughness and eliminate large-scale surface defects, the Ti64 sheets were polished using 1200-grit grinding paper, since the as-received samples contained pronounced scratches.

The SS 316L AM samples were produced in a 3D printer (TruPrint 3000, Trumpf) using the laser powder bed fusion (LPBF) process by applying thin layers of metal powder (LPW-316-AAAV, LPW technologies) to a build platform. A laser beam is then directed at specific areas of the metal powder, heating and melting the material together to form a solid layer. Afterward, the building platform is lowered to accommodate the subsequent layer. Following the printing process, the samples were depowdered and removed from the build platform. No additional post-processing was performed.

### Laser Structuring Process

Laser structuring was conducted using an optical configuration based on a two-beam interference setup (see Figure 2a). The experimental system employed a solid-state laser (Innoslab PX, EdgeWave) capable of delivering laser pulses with a pulse duration of 12 ps and a maximum average output power of 30 W. The infrared laser beam, with a wavelength λ of 1064 nm, was first expanded using a two-lens telescope system and subsequently directed into a DLIP optical head (ELIPSYS, SurFunction GmbH). This head incorporates a diffractive optical element to split the primary beam into two sub-beams, which are then shaped into elliptical profiles (see Figure 2b).

**Figure 2:**
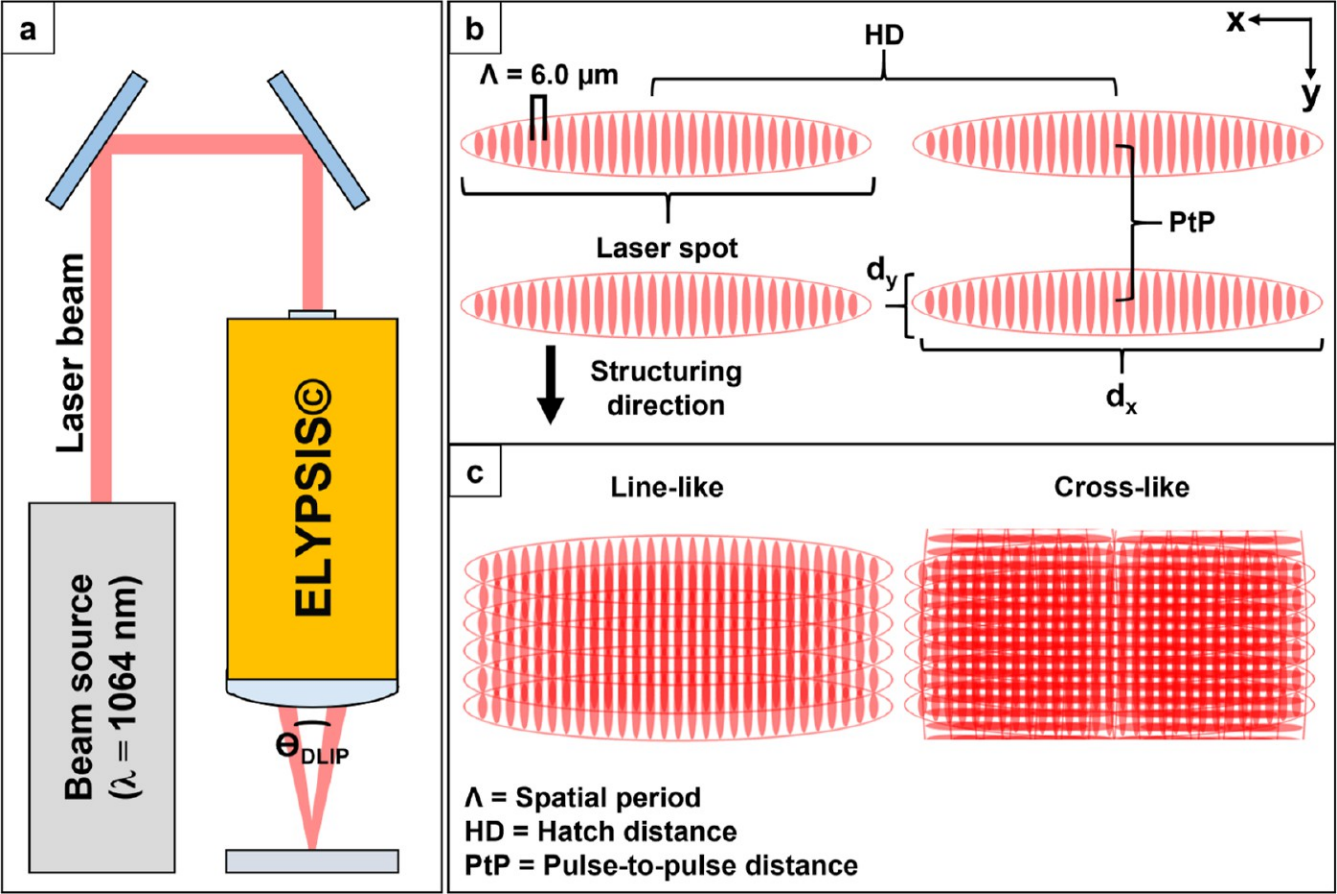
(a) Optical configuration ELYPSIS based on two beam interference optics. (b) Structuring strategy for large area processing using pulse-to-pulse distance and hatch distance. (c) Intensity distribution for line-like and cross-like DLIP features.

The optical head enables a substantial depth of focus (approximately 10 mm) and generates an elliptically shaped laser spot in the focal plane with dimensions of 1.03 ± 0.06 mm × 0.09 ± 0.01 mm (d*x* × d*y*). The spatial period Λ of the resulting interference pattern is determined by the laser wavelength λ and the angle between the two interfering beams θ_DLIP_, as given by eq 1

1 $$\Lambda =\frac{\lambda }{2\cdot \sin\frac{\theta_{DLIP}}{2}}$$

Two-dimensional movement of the metallic substrates was achieved using precision mechanical stages (PRO155–05, Aerotech). For this study, cross-like DLIP surface features with a spatial period Λ = 6.0 μm were fabricated. To generate these patterns, the substrates were initially processed to form a linear DLIP structure, rotated by 90° within the *x*–*y* plane, and then reirradiated using identical process parameters (see Figure 2c).

Surface structuring was carried out at a fixed laser repetition rate of 10 kHz and a pulse-to-pulse (PtP, see Figure 2b) spacing of 3.5 μm, using a constant single-pulse energy of 0.45 mJ, equaling a laser fluence of approximately 0.62 J cm^–2^. For large-area structuring, the hatch distance (HD)—defined as the lateral distance between adjacent scan lines—was set to 25 Λ, corresponding to HD = 150 μm.

### Topographical Characterization

The surface topography of the laser-treated samples was evaluated by confocal microscopy images (S-Neox, Sensofar) using a 50× magnification objective. Their surface profiles and average structure depths, as well as selected roughness parameters were obtained using the SensoMAP Advanced Analysis Software (Sensofar): Sa (arithmetical mean height), Sq (root-mean-square height) and Sz (maximum height), as well as the developed interfacial area ratio Sdr.

In addition, high resolution images of the treated substrates were taken by SEM (Quattro ESEM, Thermo Fischer Scientific), operating at an acceleration voltage of 12 kV.

### Wettability Characterization

The untreated substrate materials were stored in closed centrifugal tubes (15 mL, polypropylene, LABSOLUTE) and DLIP-structured samples in closed sample beakers (60 mL, polypropylene, LABSOLUTE). In each case one batch was stored filled with DI water and one batch without.

The surface was characterized by measuring the apparent static WCA (influenced by surface chemistry and surface roughness) via sessile droplet method using a contact angle measurement system (OCA 200, DataPhysics Instruments GmbH) to track the wettability development depending on the different storage media. Each measurement was performed with a drop volume of 2 μL under ambient conditions (22 °C, 40% humidity, 1003 hPa). The measurement error is described by the standard deviation of six repetition measurements. Prior to the measurements, the water-stored samples were dried with a jet of pressurized air for approximately 10 s to remove residual water.

Once the WCA stabilized after sufficiently long storage time, the OCA 200 was used to determine the SFE of the unstructured Ti64 and SS 316L substrates. The analysis was based on the OWRK method (a model developed by Owens, Wendt, Rabel and Kaelble), which divides the interfacial interactions into polar and dispersive components.^36,37^ Deionized (DI) water and diiodomethane (CAS 75-11-6, 99.0% purity, Sigma-Aldrich), with their respective values listed in Table 1, were employed as polar and dispersive test liquids and five measurements with each liquid were performed to determine the SFE.

**Table 1:** Surface Tension, Polar and Dispersive Components of the Liquids Used for the OWRK Method

liquid	surface tension (mN/m)	polar components (mN/m)	dispersive components (mN/m)
water	72.30	53.60	18.70
diiodomethane	50.80	1.30	49.50

Additionally, CAH measurements of the DLIP-treated air-stored Ti64 were performed, using a second contact angle measurement system (DSA100 Drop Shape Analyzer, Krüss) capable of tilting the entire setup. For the tilted plate method, a 5 μL droplet was placed on the substrate and the setup was gradually inclined, allowing gravity to deform the droplet shape. For each tilting step (5°, 10°, 15°, 20°, 30°, 40°, 50°), the system-specific software analyzed the WCA on each side of the droplet.

### Bubble Evolution Recordings and Image Analysis

The bubble nucleation and growth on the different substrates was captured utilizing a basic optical setup as described by Heinrich et al. in earlier publications.^35,38^ For each experiment, an O_2_-oversaturated mixture was freshly prepared, using an aluminum bottle (9393, BGS technic, 650 mL) as pressurized vessel filled with 350 mL DI water. To remove any other dissolved gases and to ensure that the nucleating bubbles mainly consisted of O_2_, the solution was flushed several times with O_2_ (99.998% purity, Air Liquide) with a final absolute pressure of 1.75 bar (see Supporting Information, Appendix A). Subsequently, the pressurized vessel was disconnected from the O_2_ pressure tank and a tube (1 mm inner diameter) was attached to the outlet valve while the other end was fixed near the bottom of the cuvette with the sample.

DLIP-treated air-stored samples formed so-called plastrons upon immersion in water—an extreme manifestation of gas-filled cavities, i.e., thin layers of air within rough protrusions on low surface energy materials, which can further be stabilized by pinning effects. Plastrons are metastable and tend to degrade over time due to the diffusion of trapped air into the surrounding water, causing a transition from the Cassie–Baxter wetting regime to the Wenzel regime. Depending on the gas saturation of the liquid, the wettability and especially the roughness factor r, plastrons can remain stable from days up to months.^39,40^ Therefore, it was crucial to remove these air layers prior to the actual experiments to ensure comparability between the different sample types.

After placing the sample, the release valve was gradually opened to allow a gentle inflow of the liquid until a filling level of 2 cm was reached. Subsequently, the optical focus was adjusted once the liquid had reached a quiescent state. The O_2_-oversaturated DI water mixture enabled the formation of bubbles that consisted of >95% O_2_.^38^ The bubble evolution was captured in gray scale images (8 bits in TIFF format), from 60 s after filling to 960 s with a frame rate of 0.1 fps and an overall viewing area of 8.6 × 7.2 mm (except for the untreated SS 316L AM with a viewing area of 4.3 × 3.6 mm^38^). For the analysis of each image, Stardist, a neural network for detecting non-overlapping star-convex objects, was applied.^41–43^ The models trained on the images of the conventional and AM substrates attribute each pixel either to a particular bubble or the image background, enabling the determination of various parameters, namely the area-equivalent circular diameter, the bubble number density and the visual surface coverage (see Supporting Information, Appendix C).

## Results and Discussion

### Topography Analysis of ps-DLIP Structures

A cross-like DLIP morphology was produced during laser structuring, as described in Section Laser Structuring Process, through material ablation and selective melting. Figure 3 shows the confocal microscopy (left panel) and SEM images (right panel) of the laser-treated substrates, illustrating the formation of periodic DLIP structures across all laser-treated surface topographies. Additionally, Table 2 summarizes the average structure depths and corresponding surface roughness parameters (Sa, Sq, Sz and Sdr) for the untreated and laser-treated metallic substrates.

**Figure 3:**
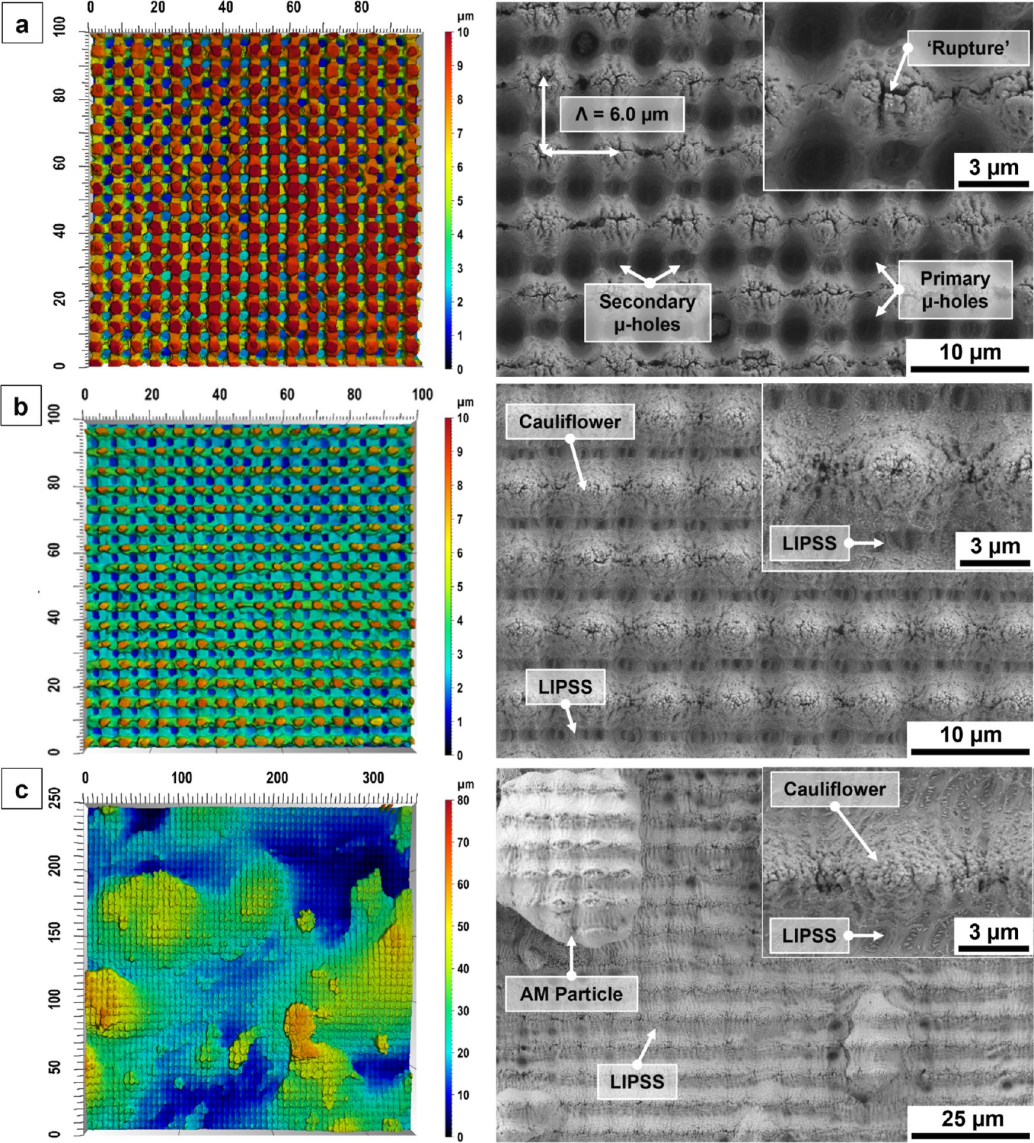
Confocal microscopy and SEM images after DLIP treatment of (a) Ti64, (b) SS 316L and (c) SS 316L AM (confocal microscopy images of the untreated samples are provided in the Supporting Information, Appendix B).

**Table 2:** Surface Parameters of Untreated and DLIP-Treated Samples, Obtained by Confocal Microscopy: Arithmetical Mean Height Sa, Root Mean Square Height Sq, Maximum Heigh Sz, Developed Interfacial Area Sdr, Roughness Factor r and Structure Depth

material	treatment	Sa (μm)	Sq (μm)	Sz (μm)	Sdr (%)	*r*	structure depth (μm)
Ti64	untreated	0.07	0.10	2.76	≈0.01	1.00	n/a
	DLIP	2.34	2.79	11.70	≈234	3.34	8.5 ± 05
SS 316L	untreated	0.36	0.47	3.22	≈2	1.02	n/a
	DLIP	1.34	1.76	8.38	≈131	2.31	6.3 ± 04
SS 316L AM	untreated	16.30	21.20	126.00	≈215	3.15	n/a
	DLIP	9.91	12.10	66.90	≈303	4.03	5.3 ± 13

From the images in Figure 3, it can be observed that the properties of the irradiated material strongly influence the resulting periodic microstructures. For instance, the magnified SEM inset in Figure 3a (right panel) for Ti64 revealed not only a cross-like DLIP pattern but also well-defined periodic μ-holes (i.e., holes or perforations emerging during the DLIP process with typical diameters in the micrometer range) located at the intersection points of the orthogonal line patterns. The primary μ-holes, with an average diameter of 2.4 ± 0.2 μm, display noticeable depth and highly circular shape. These geometrical properties are attributed to cumulative exposure and enhanced local energy absorption at the overlap regions of both irradiation steps. In contrast, the secondary μ-holes with an average diameter of approximately 1.5 ± 0.2 μm were observed along individual scan lines between intersections. Their features appeared shallower and slightly elongated, as they were formed only by the last irradiation step. The coexistence of both hole types demonstrates that the two-step laser treatment induced distinct surface morphologies, where local variations in absorbed laser fluence determine the depth and geometry of the structures.

At the structural peaks of the individual DLIP patterns (magnified inset in Figure 3a, right panel), “ruptures” and submicron roughness features can be seen, which are most likely associated with the incomplete merging of redeposited material during the structuring process, as previously reported by Ränke et al.^44^ The structures obtained on Ti64 exhibited an average structure depth of 8.5 ± 0.5 μm, which resulted in a substantial increase in Sdr of ≈234%. As shown in Figure 3b,c (left panels), the laser-treated SS 316L samples also exhibited a distinct periodic surface topography. The average structure depth of the DLIP textures was 6.3 ± 0.4 μm for the conventionally manufactured SS 316L material and 5.3 ± 1.3 μm for the AM substrate. In case of the conventional SS 316L, the DLIP treatment led to a significant increase in Sdr of ≈131%. In comparison, the untreated AM SS 316L substrate is already characterized by a very high intrinsic roughness with an initial Sdr of ≈215%, which increased further to ≈303% after the DLIP process. The corresponding SEM images in Figure 3b,c (right panel) display regular and consistent DLIP structures, which were decorated with additional LIPSS features, visible along the horizontal valleys of the surface topography. For both types of SS 316L, the emerging LIPSS were oriented perpendicular to the beam polarization used in the final processing step for creating the cross-like DLIP structures. Furthermore, for both SS 316L substrates, cauliflower-like structures were observed at interference minima positions (see magnified insets in Figure 3b,c, right panel). These consist of evenly distributed microclusters with a cone-like morphology, formed from molten particles that accumulated and resolidified at regions with the lowest laser intensity, generating highly porous structures located at structure peaks of the DLIP pattern. On the contrary, the DLIP-treated Ti64 is neither characterized by LIPSS nor by cauliflower structures. Also, particles originating from the AM process can be seen in Figure 3c (right panel). It should be noted that nanoscale features, as seen in the SEM images, cannot be properly characterized by confocal microscopy.

In general, the SS 316L substrates are characterized by reduced DLIP structure depth and different surface features compared to Ti64, despite identical laser-treatment conditions. This can primarily be attributed to differences in thermal material properties, as listed in Table 3. The higher thermal conductivity of SS 316L promotes rapid lateral heat dissipation, which lowers the local peak temperature and limits melt penetration, whereas Ti64 confines heat more effectively, leading to deeper melt pools despite its higher melting point.

**Table 3:** Thermal Substrate Properties

material	melting point (°C)	thermal conductivity (W m^–1^ K^–1^)
Ti64 ^45^	1635–1665	6–8
SS 316L^46^	1371–1399	16

### Laser-Based Wettability Modification

The wettability change after laser structuring is a complex process, influenced by several factors. Immediately after the treatment, the superficial metal oxide layer is dominated by polar components. Subsequently, various subprocesses lead to the adsorption of organic compounds onto the surface, altering the overall wettability to an extent that depends strongly on the surrounding environment. Additional insights into these phenomena are provided in the Supporting Information (Appendix E), supported by X-ray photoelectron spectroscopy (XPS).

Following the laser structuring process, the samples were stored either in ambient air or in DI water and their change in wettability was tracked for up to 140 days until a steady state was reached. As displayed in Figure 4a, untreated Ti64 and SS 316L samples exhibited moderately hydrophobic behavior when stored in air, stabilizing at WCA values between 90° and 100°. Notably, the untreated SS 316L AM reached up to 120°, caused by the inherent roughness of the material and irregular surface morphology limiting the droplet spreading. The untreated water-stored counterparts, displayed in Figure 4b, undergo a hydrophilization with final WCAs in the range of 50° to 70°. As can be seen in Figure 4c, the Ti64 substrates subjected to DLIP treatment and subsequent storage in ambient air were characterized by a superhydrophobic WCA of approximately 155° within a few hours post laser treatment. This effect has not been reported in the literature so far and at first sight contradicts with the commonly observed polar metal-oxide layer directly after laser treatment.^7,27–29,35^ Indeed, the surface properties immediately after the DLIP process may differ from the state when the first contact angle measurements are taken in this work. However, for practical reasons, a certain period of time must be allowed to pass to compare air-stored and water-stored samples. Similarly, in technological applications, there will be a certain time interval between laser structuring and the implementation of the respective part, which is better represented by the selected start of the WCA measurements. Notably, the apparently superhydrophobic character remained even after an enhanced cleaning procedure involving immersion in an ultrasonic ethanol bath (CAS 64-17-5, absolute, SUPELCO) for 90 min at 50 °C. Additional cleaning with an ultrasonic bath in acetone (CAS 67-64-1, >99.5%, Sigma-Aldrich) for 60 min at 50 °C slightly reduced the WCA to 145°. This procedure should remove any hydrophobic residues on the surface and generate a hydrophilic surface chemistry by restoring the superficial oxide layer. Since the DLIP treatment influences both surface chemistry (i.e., formation of a superficial metal oxide layer) and topography (i.e., micro- and nanostructures), the change in surface structure seems to dominate for the Ti64 substrate. The persistence of low wettability after cleaning confirms that alterations in surface topography determine the wetting behavior for this sample type. Both SS 316L and SS 316L AM reach a final WCA of roughly 100° post treatment. Therefore, the DLIP-treated SS 316L AM appears less hydrophobic compared to its non-structured reference. The water-stored counterparts (shown in Figure 4d) have a highly hydrophilic character at the start of the measurement, reaching final contact angles of 40°–50° for Ti64 and SS 316L, which are lower than those of the untreated references. Measurements for SS 316L AM were not possible due to capillary imbibition, resulting in a WCA of 0°.

**Figure 4:**
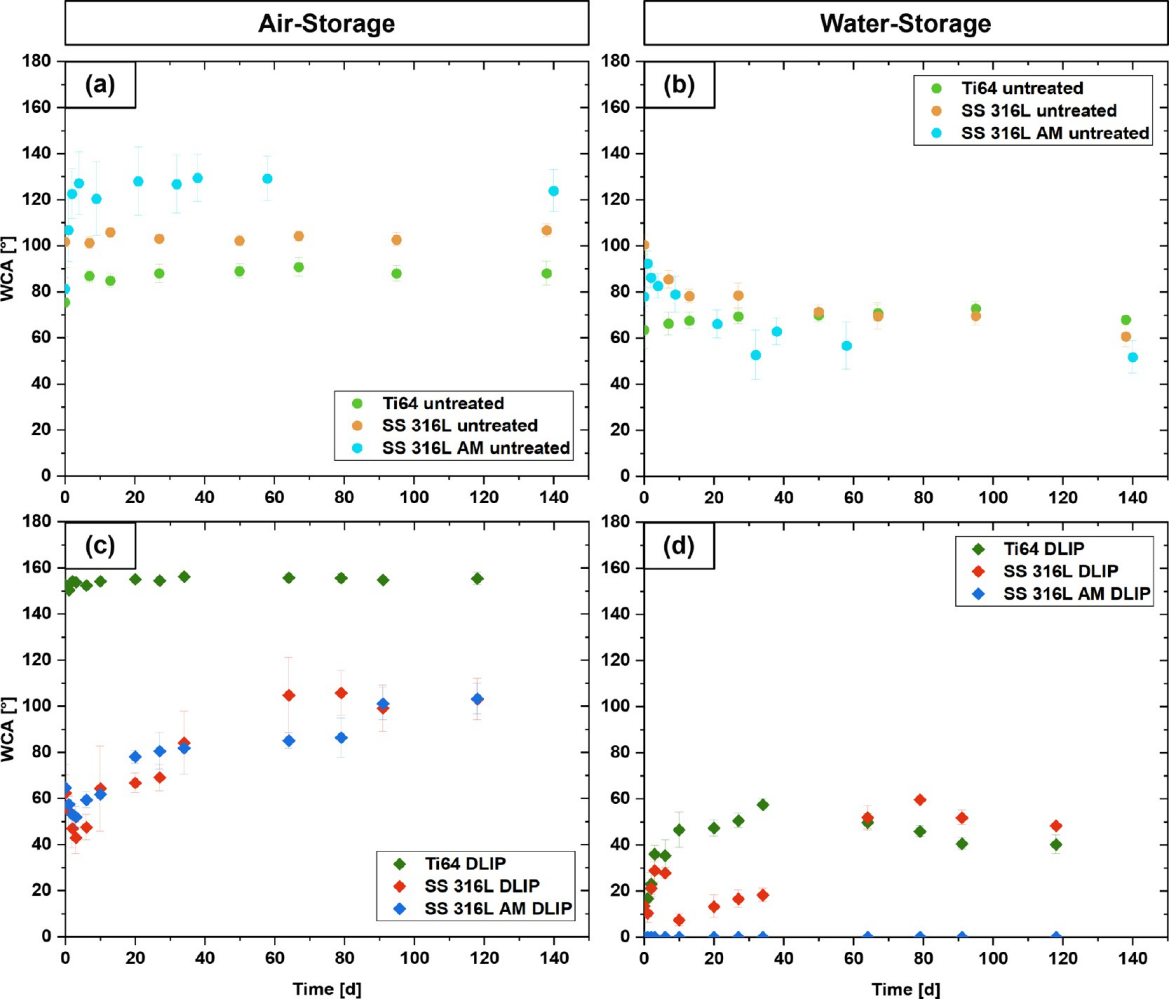
Wettability change over time for Ti64, SS 316L and SS 316L AM for (a) untreated samples stored in air, (b) untreated samples stored in water, (c) DLIP-structured samples stored in air and (d) DLIP-structured samples stored in water.

SFE measurements for the planar untreated reference materials were performed after the WCA measurements were concluded and are listed in Table 4. All air-stored samples have a lower SFE, which is dominated by the dispersive components. Similar to the DLIP-treated surfaces, it can be assumed that the surface chemistry of untreated materials also adapts to the surrounding media, but to a lower degree. In ambient air, mainly airborne species in the form of organic molecules will be adsorbed, while water-storage can also introduce additional polar groups, e.g., hydroxyl groups or the formation of hydrogen bonds with interfacial water molecules.^7,35^ Therefore, the untreated references show significantly increased polar contributions and a higher SFE due to water storage. It should be noted that the SFE determination via contact angle measurements of different test liquids is not applicable for DLIP-structured and all SS 316L AM samples due to their high surface roughness.^8,9^

**Table 4:** Surface Free Energy γ_SG_ (SFE), Polar and Dispersive Components for Untreated Samples Determined by OWRK Method

substrate	treatment	storage	SFE (mN/m)	polar (mN/m)	dispersive (mN/m)
Ti64	untreated	air	36.19 ± 0.36	2.5	33.7
	untreated	water	43.82 ± 0.39	8.8	35.0
SS 316L	untreated	air	34.76 ± 0.35	0.1	34.7
	untreated	water	44.20 ± 0.38	11.2	33.0

To better understand the surprisingly high WCA values of the DLIP-treated Ti64 surface (air-stored), CAH was measured with the tilting-plate method by recording the left and right contact angles, θ_left_ and θ_right_, displayed in Figure 5a. Because the droplet did not begin to slide at tilt angles up to 50°, the standard condition for determining CAH from θ_rec_ and θ_adv_ shortly before droplet sliding was not met.^47^ Still, the lack of sliding implies that the actual CAH exceeds the observed angle difference θ_Δ_ of >45° (Figure 5a,b). The extreme hysteresis behavior is further illustrated in Figure 5c by complete droplet adhesion at 180° surface tilt. These characteristics are indicative of the rose petal effect. On this basis, it can be assumed that water penetrates into the micro-roughness, but only partially wets the hierarchical structures, causing strong hysteresis effects, and an overall superhydrophobic WCA.^13^

**Figure 5:**
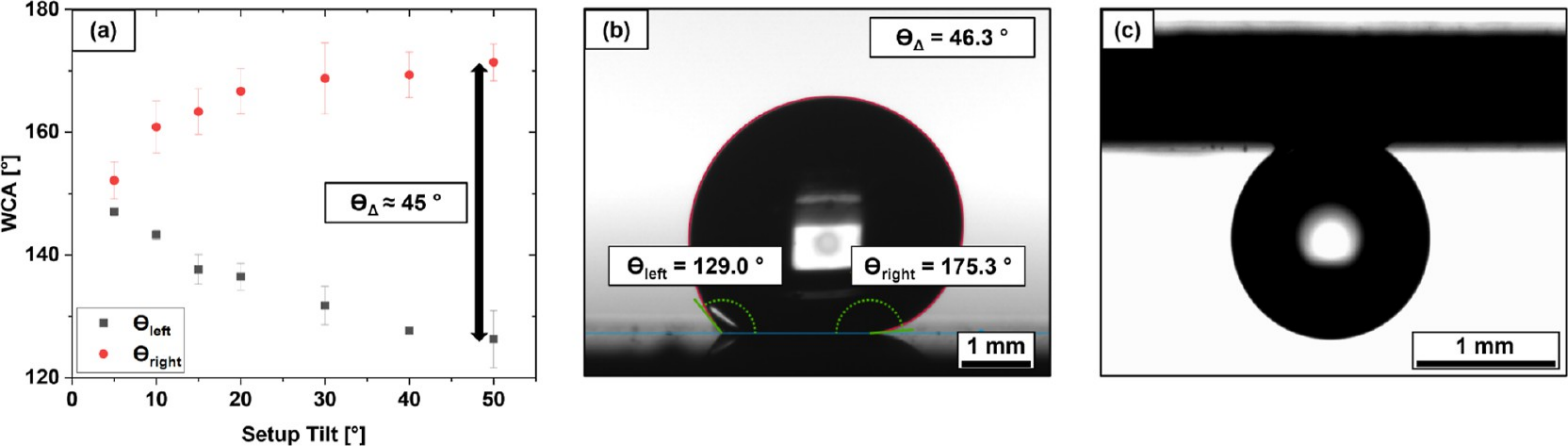
(a) WCA and θ_Δ_ depending on the overall setup tilt for DLIP-treated Ti64 stored in air, (b) θ_Δ_ measurement for a sample at 50° setup tilt and (c) droplet adhesion on the surface at 180° tilt.

### O_2_ Bubble Evolution and Growth Dynamics

The solid–gas interactions of the different substrate surfaces and dissolved O_2_ were investigated by nucleation experiments. Representative images of all 12 distinct substrate types can be seen in Figures 6 and 7 (note the different image scaling for the untreated SS 316L AM), illustrating the bubbles formed 900 s after being immersed in the O_2_-oversaturated liquid.

**Figure 6:**
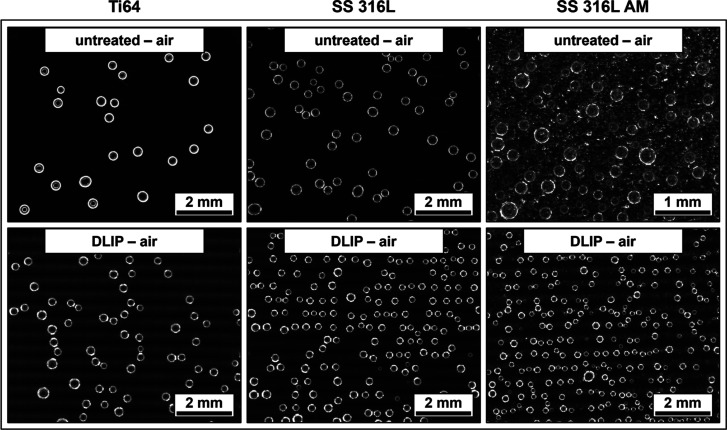
Bubble evolution at 900 s after immersion in O_2_-oversaturated solution for previously air-stored untreated and DLIP-structured samples (note the different image scaling for the untreated SS 316L AM).

**Figure 7:**
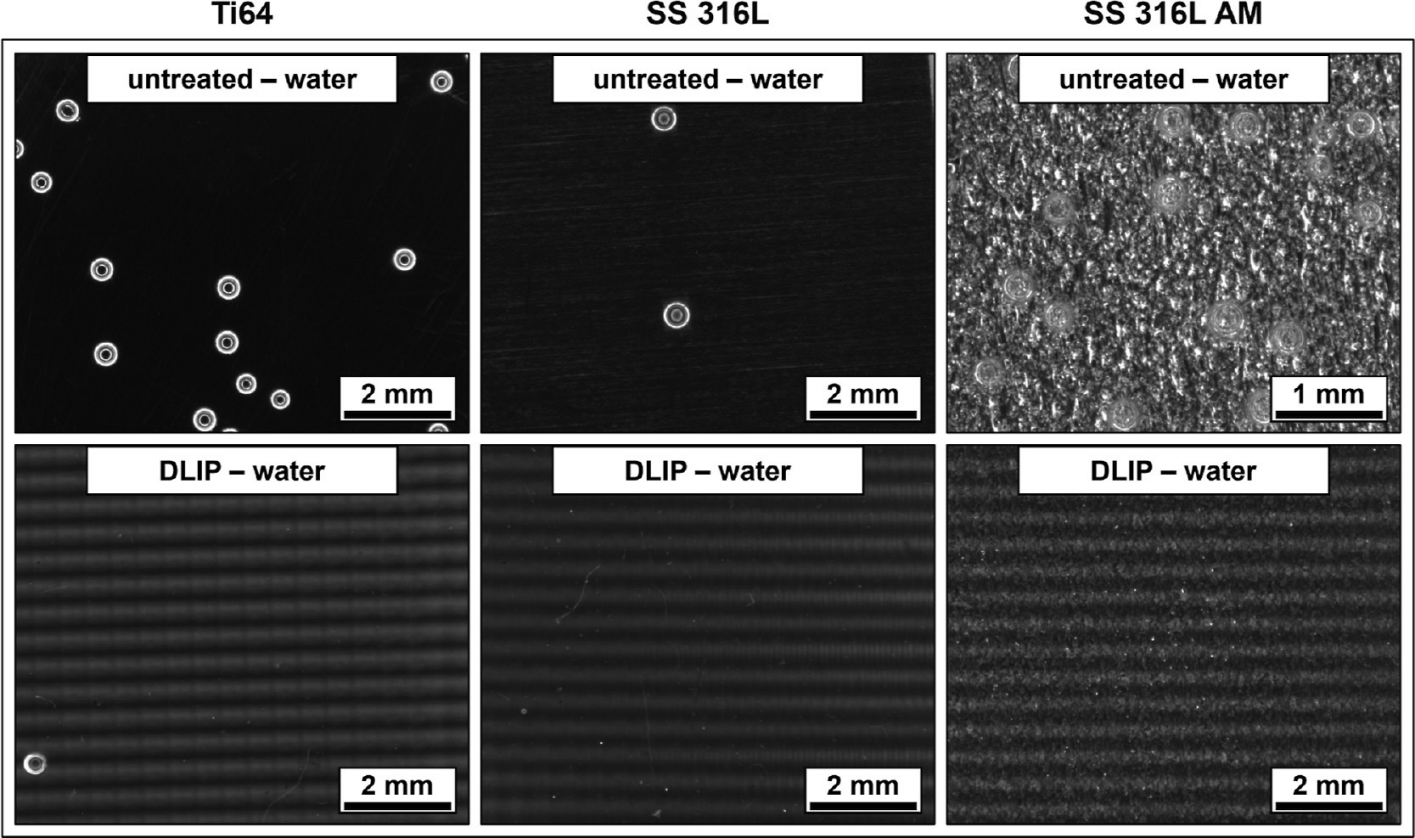
Bubble evolution at 900 s after immersion in O_2_-oversaturated solution for previously water-stored untreated and DLIP-structured samples (note the different image scaling for the untreated SS 316L AM; this specific sample type is already included in ref 38 and only listed for comparison).

When comparing both figures, the air-stored samples show enhanced solid–gas interactions in the form of bubble nucleation compared to their water-stored counterparts. On one hand, similar to the untreated references, the storage in ambient air may result in a surface free energy mostly consisting of dispersive components, causing weaker wetting and allowing for stronger interactions with the dissolved gas molecules. Additionally, upon immersion in the measurement liquid, the formation of gas cavities and Harvey nuclei cannot be completely ruled out, which promotes Type III and Type IV nucleation. The water-stored samples therefore show only very small amounts of nucleation sites, particularly in the case of DLIP-structured samples, due to their enhanced wettability. However, especially for the DLIP-treated samples with subsequent storage in air, a line-like nucleation pattern can be seen in some areas of the substrate surface, despite the applied cross-like DLIP structures. It appears in a similar spacing to the period of the visible dark/bright pattern with a significantly larger scale than the actual spatial period Λ of 6.0 μm. This pattern is caused by the HD pulse overlap of the second laser scan, producing darker lines in regions of high intensity and brighter lines in regions of low intensity.^48^

For a better quantitative comparison of the different sample types, several parameters associated with bubble evolution were analyzed: the area-equivalent circular diameter, the bubble number density and the visual surface coverage, starting from 60 s after immersion in the O_2_-oversaturated liquid (see Figure 8; note the different axis scaling between the individual figure panels). The error bars represent the standard deviation of the repeated measurements, typically based on five measurements per sample type.

**Figure 8:**
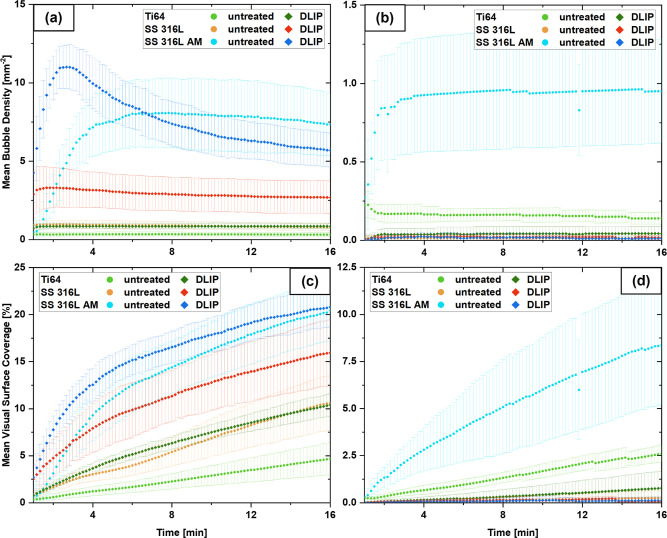
Bubble density versus the bubble evolution time for untreated and DLIP-structured samples stored in (a) air and (b) water. Visual surface coverage versus the bubble evolution time for untreated and DLIP-structured samples stored in (c) air and (d) water. (Note the different axis scaling in a,b and c,d.)

For air-stored substrates, the lowest bubble densities were observed on the untreated Ti64 and SS 316L surfaces, as well as on DLIP-treated Ti64. Despite the apparent superhydrophobicity of the DLIP-treated Ti64, the surface does not exhibit a significantly increased number of nucleation sites. A moderate increase in bubble density was observed on DLIP-treated conventional SS 316L, while the highest bubble densities appeared on both untreated and DLIP-treated SS 316L AM, attributed to the intrinsic roughness, which introduces more Harvey nuclei. Figure 8a shows that the DLIP-treated SS 316L AM samples exhibit an initial maximum in bubble density at around 2 min, followed by a decrease over time, which can be attributed to coalescence and bubble merging. In the case of water storage (note the different axis scaling between Figure 8a,b), bubble nucleation was generally suppressed for all DLIP-structured samples as well as for SS 316L. A noticeable bubble density was observed only for the untreated Ti64 and untreated SS 316L AM samples. The latter again showed the highest bubble density: its intrinsic roughness lowers the nucleation energy barrier, but the surface remains less hydrophilic than SS 316L AM DLIP, whose surface chemistry was strongly modified by the laser structuring process.

However, bubble density alone is not sufficient to characterize the solid–gas interactions, as bubble size can vary significantly. The bubble diameter diagrams (see Supporting Information, Appendix D) show that the detection based on Stardist can characterize the first bubbles at roughly 90 μm. Across the different sample types, the bubble diameters range from 190 to 490 μm at the end of the recording. Bubble density and bubble diameter are inversely related: high bubble densities lead to smaller bubbles. During mass transfer from the oversaturated solution, individual bubble growth becomes diffusion-limited, as neighboring bubbles compete for the locally available dissolved O_2_. Surface coverage, incorporating both bubble density and diameter, provides a more effective basis for comparison. Among the air-stored samples (see Figure 8c), the surface coverage of the untreated materials followed a trend consistent with their WCA (Ti64 < SS 316L < SS 316L AM). Surface coverage was higher for all DLIP-treated variants, with the apparently superhydrophobic Ti64 displaying the weakest solid–gas interactions. By the end of the recording, both untreated and DLIP-structured SS 316L AM reach >20% surface coverage. Initially, surface coverage increases faster for the DLIP-treated substrate owing to its additional micro- and nanostructures and larger interface area. In contrast, the water-stored samples (see Figure 8d) generally exhibited lower surface coverage across all materials.

As highlighted in Table 4, the hydrophilization induced by the aqueous storage increases SFE and polar contributions, resulting in a higher bubble nucleation energy barrier, thereby reducing solid–gas interactions. The hydrophobization that occurs for the air-stored samples has the opposite effect. Notably, among the water-stored substrates, untreated SS 316L AM maintained the highest surface coverage. Under these conditions, DLIP-treated samples generally exhibited weaker solid–gas interactions than their untreated counterparts.

### Discussion

As observed for the planar reference samples, storage in air results in more hydrophobic surfaces dominated by dispersive components. In contrast, storage in water leads to an increase in polar contributions, resulting in higher total SFE and, consequently, more hydrophilic surface properties. These findings are in agreement with other studies.^7,27,35,49^

Table 5 attributes the assumed wetting states to the corresponding sample types and parameters. The untreated reference samples are characterized by a rather homogeneous and planar surface, therefore a Young-like wetting behavior can be assumed. The DLIP-structured Ti64 and SS 316L samples can be related to Wenzel or Cassie–Baxter states. Prolonged storage in water should remove any gas cavities and prevent Harvey nuclei, resulting in a completely wetted two-phase solid–liquid interface. On the contrary, air storage should facilitate gas entrapment. The only exception is the air-stored DLIP-structured Ti64, which exhibits the rose petal state, as described before. Lastly, no defined wetting states are assigned to the SS 316L AM substrates, since their surfaces are governed by a combination of surface thermodynamics and capillary effects due to the high intrinsic roughness and porosity.

**Table 5:** Parameter Overview

substrate	treatment	storage	*r*	WCA (°)	presumable state	SFE (mN/m)	coverage (%)
Ti64	untreated	air	1.00	88.0	Young-like	36	4.8
	untreated	water	1.00	68.0	Young-like	44	2.7
	DLIP	air	3.34	155.4	rose petal	N/A	10.7
	DLIP	water	3.34	40.2	Wenzel	N/A	0.8
SS 316L	untreated	air	1.02	106.6	Young-like	35	11.0
	untreated	water	1.02	60.7	Young-like	44	0.3
	DLIP	air	2.31	103.0	Cassie–Baxter	N/A	16.2
	DLIP	water	2.31	48.3	Wenzel	N/A	0.1
SS 316L AM	untreated	air	3.15	123.8	N/A	N/A	19.8
	untreated	water	3.15	51.7	N/A	N/A	8.9
	DLIP	air	4.03	103.3	N/A	N/A	21.1
	DLIP	water	4.03	0.0	N/A	N/A	0.1

The DLIP treatment “activates” the surface due to superficial oxidation, making it more susceptible to surface processes in interaction with the surrounding media. Additionally, the treatment introduces new hierarchical surface structures that remain sensitive to environmental exposure and can modulate the functional wetting behavior. In the context of the Wenzel and Cassie–Baxter models, the combination of surface chemistry and increased roughness can be leveraged to amplify inherent hydrophilic or hydrophobic tendencies. For AM materials, the DLIP treatment has a limited effect on bubble nucleation for subsequent storage in air, as both untreated and DLIP-treated SS 316L AM samples exhibit comparable surface coverage. However, when stored in water, the DLIP-structured AM surfaces become superhydrophilic and strongly suppress bubble nucleation. This behavior can be attributed to the hydrophilization of the generated micro- and nanostructures in the aqueous environment, which promotes capillary wicking in the narrow spaces.^50–52^ As a result, Harvey nuclei are inhibited and solid–gas interactions decrease. This leads to decreased bubble nucleation and even lower surface coverage than that of the untreated counterparts in the Young-like state. Interestingly, the rose petal effect has only a negligible influence on bubble nucleation, despite the apparent superhydrophobic WCA. For example, DLIP-structured Ti64 with a WCA of 155.4° shows a surface coverage of only 10.7%, whereas SS 316L DLIP with a lower WCA of 103.0° reaches 16.2%. This indicates that, although apparently superhydrophobic, the O_2(aq)_ to O_2(g)_ phase transition is not favored. Upon immersion, the hierarchical structures become partly wetted, leading to high CAH. Thus, it is assumed that only the nanostructures provide small cavities, and Type III nucleation dominates. In contrast, the Cassie–Baxter state appears more effective regarding solid–gas interactions, as its larger gas pockets, as illustrated in Figure 1, promote more pronounced Harvey nuclei and therefore more Type IV nucleation events, despite exhibiting a lower apparent WCA.

The overview in Table 5 highlights that WCA-based wettability measurements alone are insufficient to predict nucleation tendencies on different substrate types. A thorough understanding of surface topography, including micro-/nanostructures and overall roughness is essential, as multiple surface properties collectively govern the transition of molecular oxygen from the aqueous phase O_2(aq)_ to the gaseous phase O_2(g)_.

## Conclusion

This study investigated the modification of surface wettability resulting from DLIP structuring of Ti64, SS 316L and SS 316L AM substrates. Based on surface topography and the developed surface hydrophilicity/hydrophobicity due to the respective storage conditions, different wetting regimes were identified and their effects on O_2_ bubble nucleation behavior were analyzed. The key findings are summarized as follows:

- AM materials are characterized by stronger solid–gas interactions compared to conventional substrates, due to their intrinsic roughness, which lowers the bubble nucleation energy barrier and allows for additional Harvey nuclei.
- DLIP treatment amplifies solid–gas interactions for all substrates for storage in air, with exception of DLIP-structured SS 316L AM compared to its untreated reference. For storage in aqueous media, the opposite effect takes place, resulting in strongly reduced solid–gas interactions due to surface oxidation and hydrophilization during the laser-structuring.
- The rose petal effect primarily arises from unique topographical features and is characterized by partial wetting of the surface structures. Its nucleation behavior reveals that a superhydrophobic WCA alone is not sufficient to facilitate solid–gas interactions.

Concluding, the changes in surface chemistry and topography due to DLIP structuring strongly influence wettability and solid–gas interactions. Depending on the chosen substrate and DLIP parameters, a wide variety of wetting states can be achieved. For instance, increasing the DLIP structure depth compared to previous studies produced rose-petal surfaces with distinct wettability behavior. The combination of laser structuring with the intrinsic roughness of AM materials introduces an additional hierarchical level in the surface topography. This flexible control of surface structures opens new opportunities for optimizing bubble management in diverse technological applications.

## Supplementary Material



## Notes

O_2_ oversaturation procedure and setup, confocal microscopy images, stardist image analysis and model evaluation, bubble diameter diagrams, XPS analysis (PDF)
