# Supplementary material for: Direct Laser Interference Patterning for Wettability Modification and Bubble Nucleation on Conventional and Additively Manufactured Metals
Source: Langmuir. 2026 Mar 27;42(14):9951–64. doi: 10.1021/acs.langmuir.6c00056 (PMC13085804; doi:10.1021/acs.langmuir.6c00056)
Supplement: Supplementary file 1 [file la6c00056_si_001.pdf]

Supporting Information
Direct Laser Interference Patterning for Wettability Modification and Bubble Nucleation on Conventional and Additively Manufactured Metals

Julian Heinrich,[*,†,‡] Fabian Ränke,[¶] Karin Schwarzenberger,[†,‡] Tine Marquardt,[†,‡] Xuegeng Yang,[†,‡] Mateusz Marek Marzec,[§] Krystian Sokołowski,[§] Andrés Fabián Lasagni,[¶,‖] and Kerstin Eckert[†,‡]

†*Institute of Fluid Dynamics, Helmholtz-Zentrum Dresden-Rossendorf, Bautzner Landstr. 400, 01328 Dresden, Germany*

‡*Institute of Process Engineering and Environmental Technology, Technische Universität Dresden, Helmholtzstr. 10, 01069 Dresden, Germany*

¶*Institute of Manufacturing, Technische Universität Dresden, George-Baehr-Str. 3c, 01069 Dresden, Germany*

§*Academic Centre for Materials and Nanotechnology, AGH University of Krakow, Av. Mickiewicza 30, 30-059 Krakow, Poland*

‖*Fraunhofer Institute for Material and Beam Technology IWS, Winterbergstraße 28, 01277 Dresden, Germany*

E-mail: j.heinrich@hzdr.de

Appendix A

O_2 Oversaturation

The oversaturation was achieved by the following steps:

- Fill the pressure vessel with 350 ml DI water; connect it to the O_2 pressure tank
- Apply an O_2 overpressure of 3 bar (absolute pressure)
- Shake the vessel by hand for 3 s and afterwards open the release valve for 1 s (repeat 5 times in total); afterwards let the vessel rest for 1 min
  - Repeat the whole procedure 3 times in total
- Apply an O_2 overpressure at 1.75 bar (absolute pressure)
- Shake the vessel by hand for 3 s and afterwards open the release valve for 1 s (repeat 5 times in total); let the vessel rest for 1 min
  - Repeat the whole procedure 2 times in total

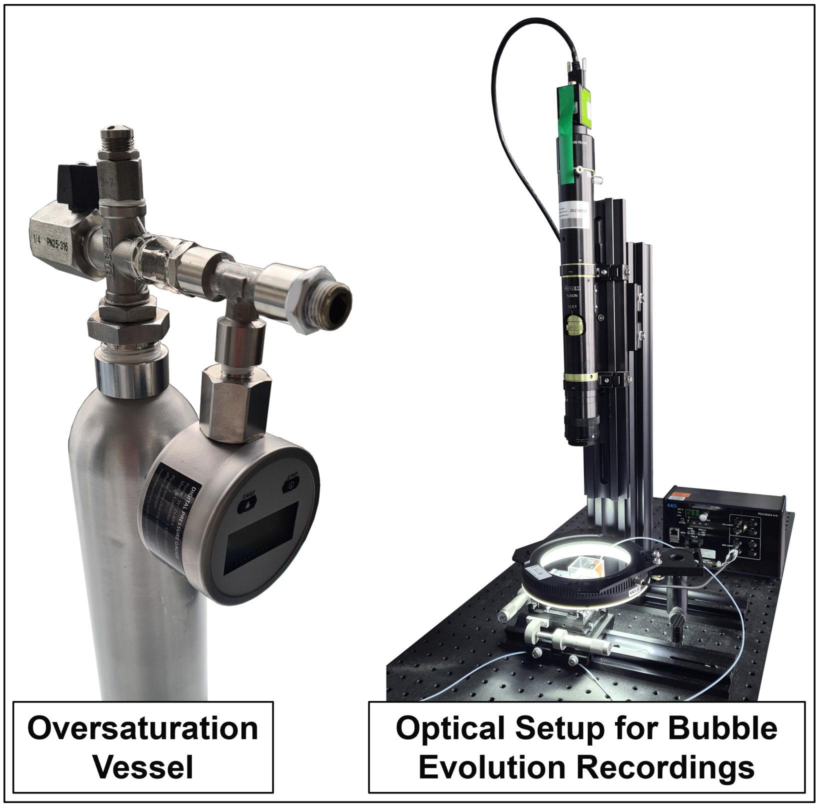

Figure S1: Oversaturation vessel and the optical setup used for bubble evoluation recordings.

More details on the oversaturation procedure as well as additional information regarding theoretical and actual O_2 concentration can be found in Ref. 1,2.

Appendix B

Additional Confocal Microscopy Images

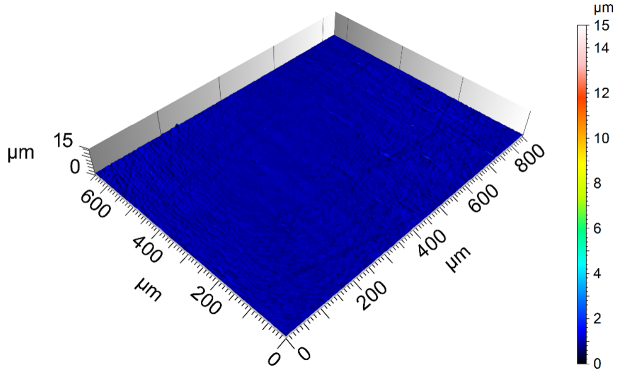

(a) Ti64 surface.

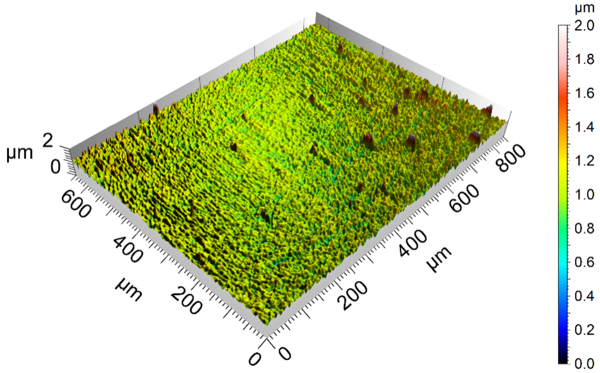

(b) Ti64 surface.

Figure S2: Confocal microscopy of untreated Ti64 with a scale of (a) 15 µm and (b) 2 µm.

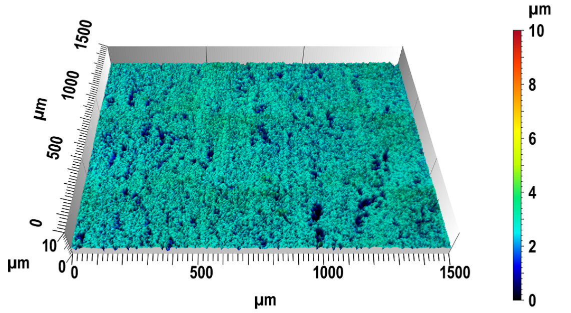

(a) SS 316L surface.

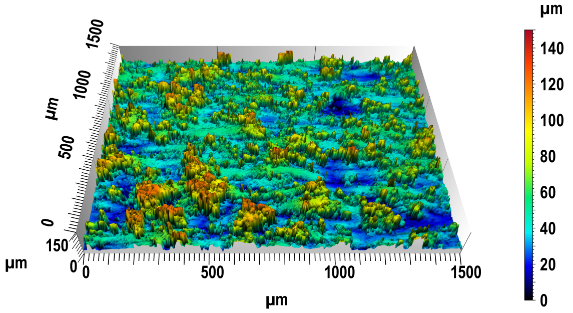

(b) SS 316L AM surface.[†]

Figure S3: Confocal microscopy of (a) untreated SS 316L and (b) untreated SS 316L AM.

[†]The image was originally published in the SI of Ref. 2 by Heinrich et al.

Appendix C

Stardist Image Analysis

To achieve a high quality of bubble recognition despite the varying image backgrounds and bubble sizes, Stardist was employed as a neural-network based method for bubble detection.[3–5] Stardist for bubble detection in electrolysis processes was already employed by Rox et al.,[5] and the code was further adapted by Heinrich et al.,[2] where more detailed information about the general bubble recognition process are provided.

The training data for Stardist was generated using the open software QuPath (v. 0.5.1).[6] The bubbles were manually annotated on image sections with a size of 1024 x 1024 pixel and the associated masks generated.

Two different models were trained: the model 'Untreated Samples' for the bubble recognition on the untreated substrates and 'DLIP Samples' for the DLIP structured surfaces. For the model 'Untreated Samples', the number of annotated training images was 24, and 10 additional images were used for model evaluation. For the model 'DLIP Samples', 34 images were annotated as training data and another 10 images were used to evaluate the model. This number of images allowed to provide a sufficient variety in bubble sizes and shapes for the training data. For the untreated SS 316L AM samples, the bubble recognition was based on the model 'SS 316L' described by Heinrich et al.[2] Figures S4 – S8 show examples of a raw image, the labeled annotations and the generated mask for each sample type after various storage conditions and different recording times.

For the training, all network architectures and training hyper-parameters were set to their default values.[3] The grid size was modified to [2,2] in order to account for the median bubble sizes. Stardist approximates detected bubble instances by star-convex polygons with a fixed number of radial directions, also named rays, which affects both the quality of shape reconstruction and the required time for instance prediction. A number of 32 rays was identified as an optimum between calculation time and quality and used for all models.

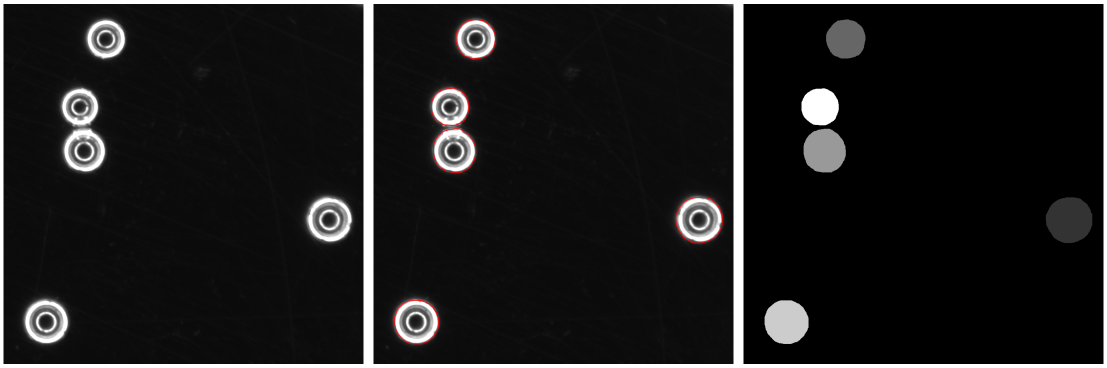

(a) (b) (c)

Figure S4: (a) Raw and (b) annotated image with (c) generated mask for untreated Ti64.

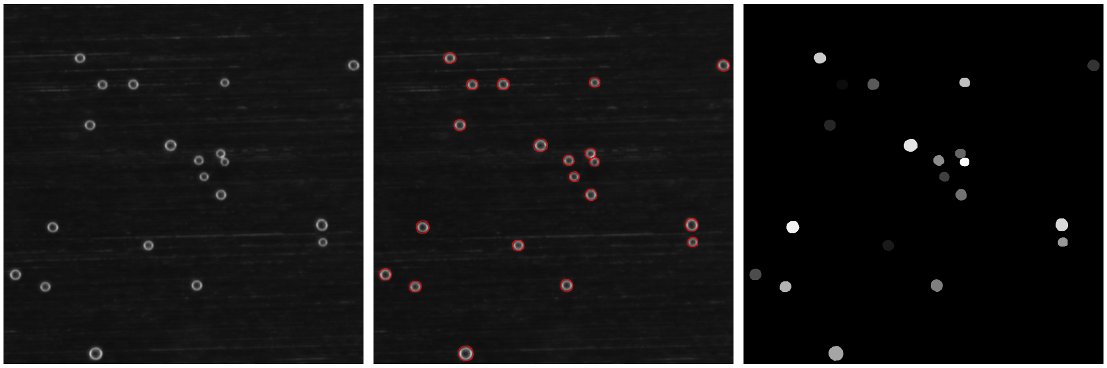

(a) (b) (c)

Figure S5: (a) Raw and (b) annotated image with (c) generated mask for untreated SS 316L.

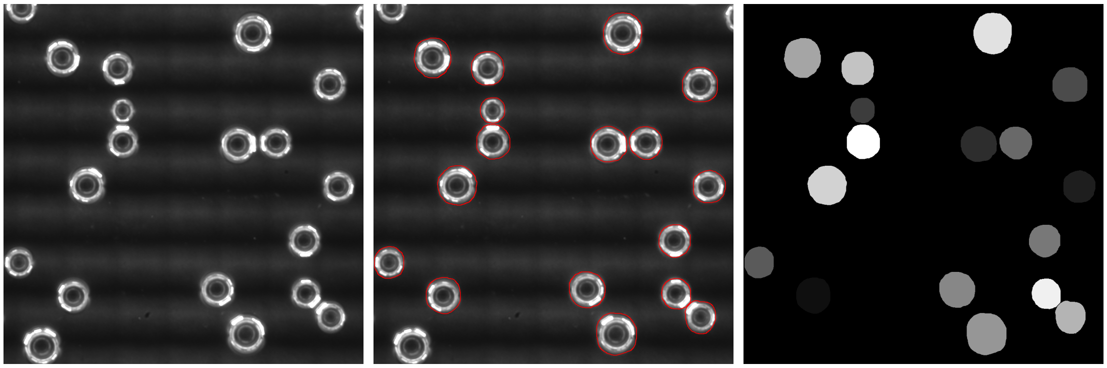

(a) (b) (c)

Figure S6: (a) Raw and (b) annotated image with (c) generated mask for DLIP-treated Ti64.

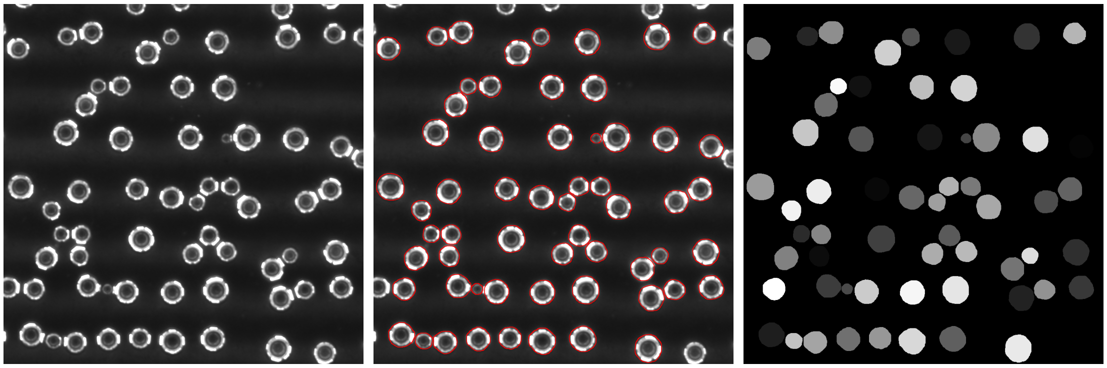

(a) (b) (c)

Figure S7: (a) Raw and (b) annotated image with (c) generated mask for DLIP-treated SS 316L.

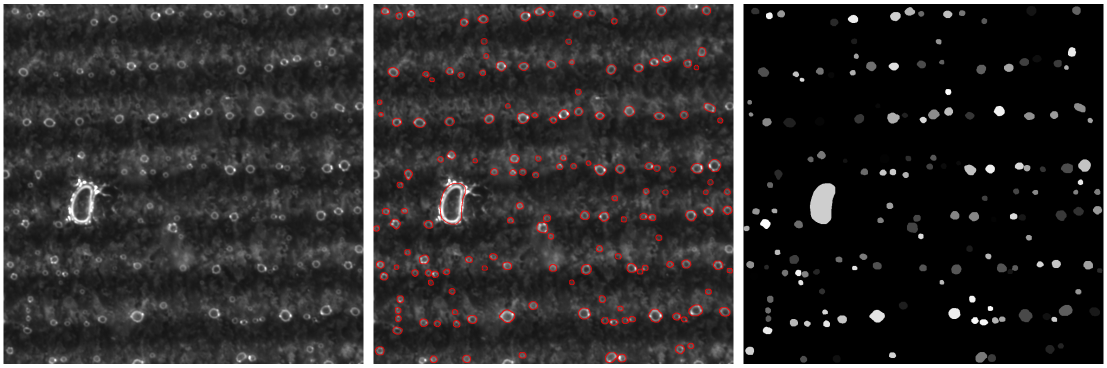

(a) (b) (c)

Figure S8: (a) Raw and (b) annotated image with (c) generated mask for DLIP-treated SS 316L AM.

Model Evaluation, Limits and Errors of Stardist

The prediction quality of image segmentation models can be evaluated using the Intersection over Union (IoU) metric, which quantifies the amount of overlap between the bubble instances on the annotated test images and the predicted objects. Bubble detections were classified as true-positive (TP), false positive (FP) and false-negative (FN) at specific IoU thresholds. Then, precision, recall, and F1 score metrics were calculated at different IoU thresholds using the following equations S1 – S3:[7]

$$\text{Precision} = \frac{TP}{TP + FP} \tag{S1}$$

$$\text{Recall} = \frac{TP}{TP + FN} \tag{S2}$$

$$\text{F1} = 2 \cdot \frac{\text{Precision} \cdot \text{Recall}}{\text{Precision} + \text{Recall}} \tag{S3}$$

The evaluation results presented in Figure S9 demonstrate very good model qualities. For an IoU of 0.8, precision and recall are above 90 % for all models. This highlights that the model correctly identifies the bubble positions and predicts their size and shape with a high accuracy, which is important for estimating the bubble metrics.

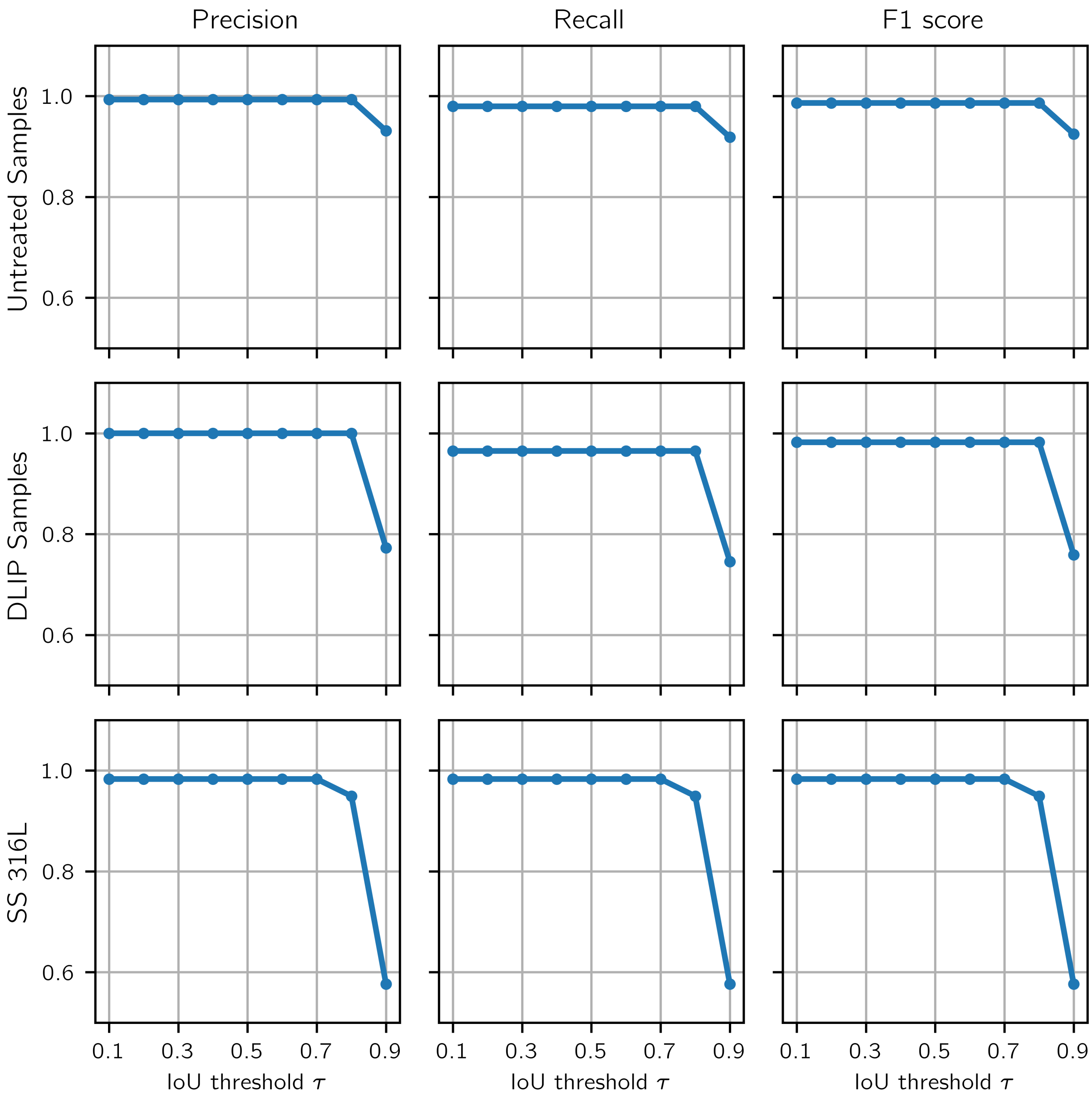

Figure S9: Evaluation of Stardist models.

Image Analysis Parameters

The used bubble metrics are visualized in Figure S10. The area-equivalent circular diameter d_{eq} was calculated for each individual bubble. Then, for each image, the overall bubble number density ρ_n and visual surface coverage VC were determined. Some bubbles are only partially visible on the image and in this context not considered for further analysis, because their reduced area and cut form would lead to a false estimation of the mean bubble diameter. To correct the ρ_n and VC for the neglected bubbles on the image edge, the overall image area was reduced by an outline with a thickness of 0.5 times the mean d_{eq} of the image, resulting in the corrected image area A_{corr}.

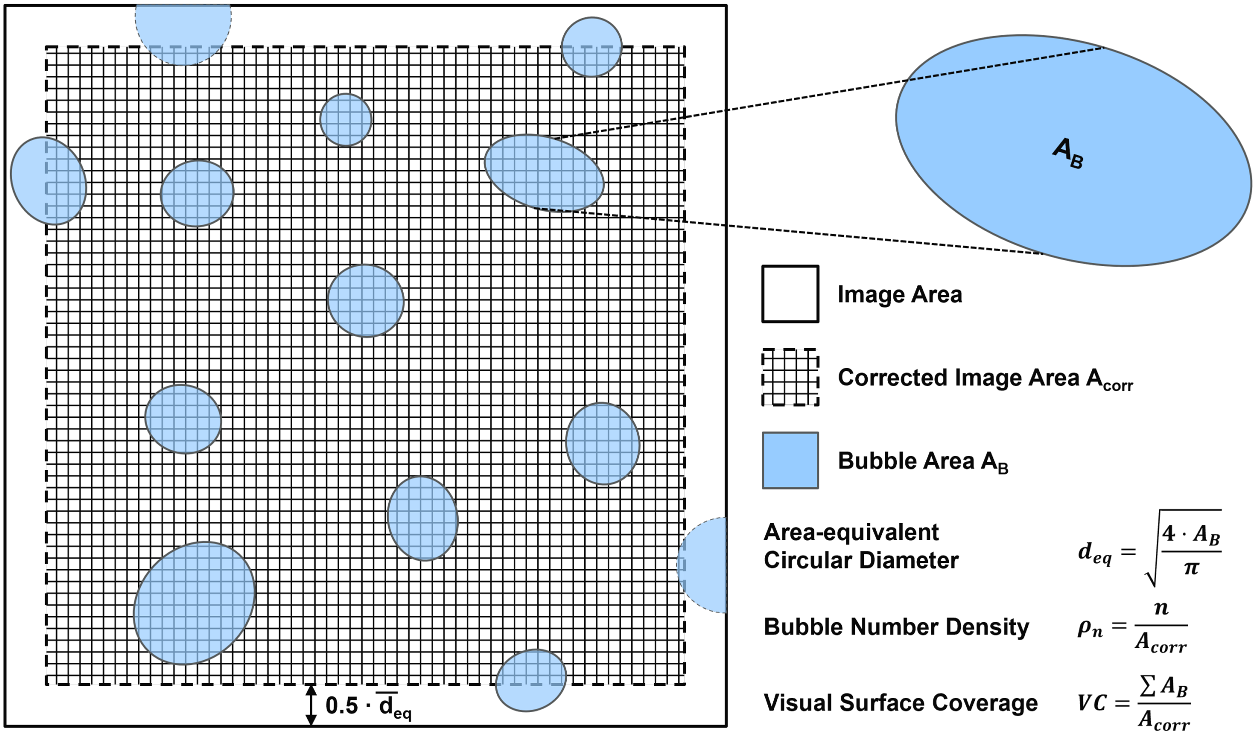

Figure S10: Visualization of analyzed bubble parameters via Stardist.

Appendix D

Bubble Evolution Analysis

Figures S11 and S12 show the bubble diameters based on the Stardist evaluation.

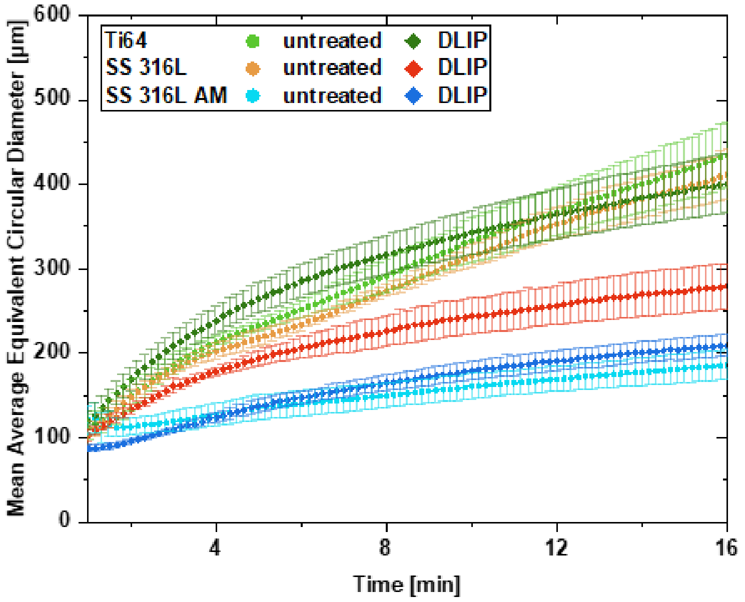

Figure S11: Bubble diameter evolution for air-stored samples.

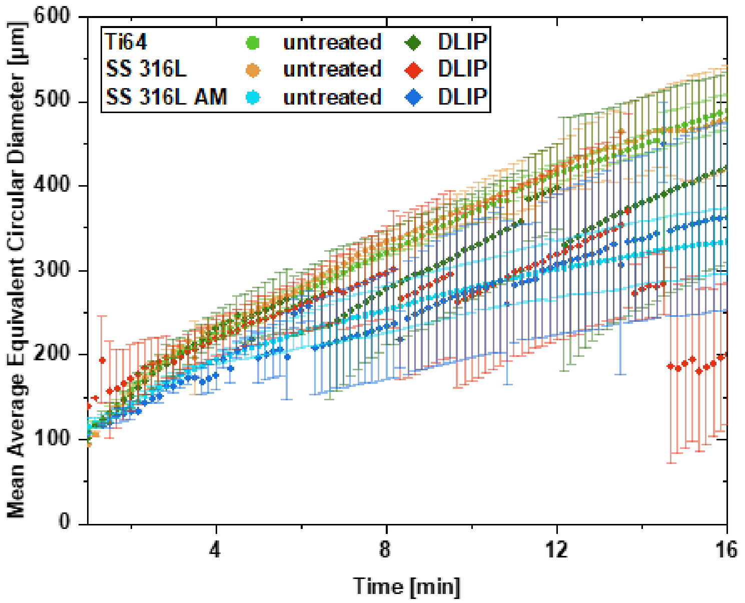

Figure S12: Bubble diameter evolution for water-stored samples.

Appendix E

XPS Analysis

Upon prolonged exposure to ambient air, a hydrophobization process occurs for previously laser-structured metal substrates, due to the adsorption of organic molecules including long-chain hydrocarbons with carbonyl groups, carboxylic acids, and diol groups. This alters the surface chemistry by enrichment of -CH_2- and -CH_3 but also -C-O-, -C=O and -COO^-, depending on the surrounding concentration and composition of airborne species.[8] Another study showed by the example of laser-treated Ti64, that also an adsorption process took place in aqueous media, mainly resulting in higher C (at. %) and N (at. %) as well as additional hydroxyl groups compared to untreated references.[1]

XPS measurements were conducted using a PHI5000 VersaProbe II (ULVAC-PHI) system with a monochromatic Al Kα as source. Each measurement was performed for an area of 400 x 400 µm and at 45° angle. Molecules down to a depth of 5 nm contribute to the XPS measurement signal; therefore, the data represents the composition near the solid surface.

It should be noted that all analysis results for the untreated SS 316L AM samples have already been published before in Ref. 2 and are only listed for comparison.

Due to the very long storage times and sample handling required for WCA measurements, the XPS values proved to be highly error prone, with large deviations between samples of the same type. Therefore, no exact values of the surface composition can be stated and instead brief estimates are given, listed in the Table S1, highlighting following trends for all samples:

- DLIP treated samples compared to the untreated substrates typically exhibit a higher O at.%, originating from the formation of a superficial laser-induced oxide layer when exposing the samples to the high intensities provided by these laser pulses in air environment.[8,9]
- All water-stored samples exhibit higher amount of C at.%.
- All samples show impurities in form of other elements in the range of up to 18 %, highlighting the adsorption of various organic compounds from the ambient media.

Table S1: XPS values (average values in case of multiple measurements with deviations up to > 15 %).

Substrate	Treatment	Storage	C (at. %)	O (at. %)	Others (at. %)
Ti64	untreated	air	61	30	9
	untreated	water	77	16	7
	DLIP	air	46	38	16
	DLIP	water	49	37	14
SS 316L	untreated	air	65	27	8
	untreated	water	70	22	8
	DLIP	air	32	51	17
	DLIP	water	58	31	11
SS 316L AM	untreated	air	40	45	15
	untreated	water	54	33	13
	DLIP	air	29	53	18
	DLIP	water	56	34	10

References

(1) Heinrich, J.; Ränke, F.; Schwarzenberger, K.; Yang, X.; Baumann, R.; Marzec, M.; Lasagni, A. F.; Eckert, K. Functionalization of Ti64 via Direct Laser Interference Patterning and Its Influence on Wettability and Oxygen Bubble Nucleation. *Langmuir* **2024**, *40*, 2918–2929.

(2) Heinrich, J.; Schwarzenberger, K.; Marquardt, T.; Yang, X.; Marzec, M. M.; Manthey, J.; Stadler, M.; Ammann, T.; Schatz, K.; Weidinger, I. M.; Eckert, K. Surface Functionalization of Additively Manufactured Polypropylene and Stainless Steel 316L: Impact on Wettability and Oxygen Nucleation. *ACS Applied Engineering Materials* **2025**, *3*, 3624–3638.

(3) Schmidt, U.; Weigert, M.; Broaddus, C.; Myers, G. Cell Detection with Star-Convex Polygons. Medical Image Computing and Computer Assisted Intervention - MICCAI 2018 - 21st International Conference, Granada, Spain, September 16-20, 2018, Proceedings, Part II. 2018; pp 265–273.

(4) Hessenkemper, H.; Starke, S.; Atassi, Y.; Ziegenhein, T.; Lucas, D. Bubble Identification From Images With Machine Learning Methods. *International Journal of Multiphase Flow* **2022**, *155*, 104169.

(5) Rox, H.; Bashkatov, A.; Yang, X.; Loos, S.; Mutschke, G.; Gerbeth, G.; Eckert, K. Bubble Size Distribution and Electrode Coverage at Porous Nickel Electrodes in a Novel

3-Electrode Flow-Through Cell. *International Journal of Hydrogen Energy* **2023**, *48*, 2892–2905.

(6) Bankhead, P.; Loughrey, M. B.; Fernández, J. A.; Dombrowski, Y.; McArt, D. G.; Dunne, P. D.; McQuaid, S.; Gray, R. T.; Murray, L. J.; Coleman, H. G.; James, J. A.; Salto-Tellez, M.; Hamilton, P. W. QuPath: Open Source Software for Digital Pathology Image Analysis. *Scientific Reports* **2017**, *7*, 16878.

(7) Géron, A. *Hands-On Machine Learning with Scikit-Learn, Keras & TensorFlow, 2nd Edition*; O'Reilly Media: Sebastopol, CA, 2019.

(8) Samanta, A.; Wang, Q.; Shaw, S. K.; Ding, H. Roles of Chemistry Modification for Laser Textured Metal Alloys To Achieve Extreme Surface Wetting Behaviors. *Materials & Design* **2020**, *192*, 108744.

(9) Florian, C.; Déziel, J.-L.; Kirner, S. V.; Siegel, J.; Bonse, J. The Role of the Laser-Induced Oxide Layer in the Formation of Laser-Induced Periodic Surface Structures. *Nanomaterials* **2020**, *10*.
